# Identification of Aminoglycoside and β-Lactam Resistance Genes from within an Infant Gut Functional Metagenomic Library

**DOI:** 10.1371/journal.pone.0108016

**Published:** 2014-09-23

**Authors:** Fiona Fouhy, Lesley A. Ogilvie, Brian V. Jones, R. Paul Ross, Anthony C. Ryan, Eugene M. Dempsey, Gerald F. Fitzgerald, Catherine Stanton, Paul D. Cotter

**Affiliations:** 1 Teagasc Food Research Centre, Moorepark, Fermoy, Cork, Ireland; 2 School of Microbiology, University College Cork, Cork, Ireland; 3 School of Pharmacy and Biomolecular Sciences, University of Brighton, Brighton, East Sussex, United Kingdom; 4 Queen Victoria Hospital NHS Foundation Trust, East Grinstead, West Sussex, United Kingdom; 5 Alimentary Pharmabiotic Centre, Cork, Ireland; 6 Department of Paediatrics and Child Health, University College Cork, Cork, Ireland; 7 Department of Neonatology, Cork University Maternity Hospital, Cork, Ireland; Agricultural Research Service, United States of America

## Abstract

The infant gut microbiota develops rapidly during the first 2 years of life, acquiring microorganisms from diverse sources. During this time, significant opportunities exist for the infant to acquire antibiotic resistant bacteria, which can become established and constitute the infant gut resistome. With increased antibiotic resistance limiting our ability to treat bacterial infections, investigations into resistance reservoirs are highly pertinent. This study aimed to explore the nascent resistome in antibiotically-naïve infant gut microbiomes, using a combination of metagenomic approaches. Faecal samples from 22 six-month-old infants without previous antibiotic exposure were used to construct a pooled metagenomic library, which was functionally screened for ampicillin and gentamicin resistance. Our library of ∼220Mb contained 0.45 ampicillin resistant hits/Mb and 0.059 gentamicin resistant hits/Mb. PCR-based analysis of fosmid clones and uncloned metagenomic DNA, revealed a diverse and abundant aminoglycoside and β-lactam resistance reservoir within the infant gut, with resistance determinants exhibiting homology to those found in common gut inhabitants, including *Escherichia coli*, *Enterococcus* sp., and *Clostridium difficile*, as well as to genes from cryptic environmental bacteria. Notably, the genes identified differed from those revealed when a sequence-driven PCR-based screen of metagenomic DNA was employed. Carriage of these antibiotic resistance determinants conferred substantial, but varied (2–512x), increases in antibiotic resistance to their bacterial host. These data provide insights into the infant gut resistome, revealing the presence of a varied aminoglycoside and β-lactam resistance reservoir even in the absence of selective pressure, confirming the infant resistome establishes early in life, perhaps even at birth.

## Introduction

There is growing concern that we are rapidly approaching a post-antibiotic era. As a result every effort is being made to discover and investigate antibiotic resistance reservoirs with the aim of limiting the selection for, or dissemination of, antibiotic resistance genes. One such reservoir is the human gut microbiota. Colonized by trillions of bacteria representing hundreds of different species, this ecosystem has been identified as a source of antibiotic resistant bacteria [1]–[3]. Though vastly less complex and considerably less stable than the adult gut microbiota, the gut microbiota of infants also has the potential to acquire and disseminate antibiotic resistant genes [4]. From the commencement of labour, the sterile infant gut rapidly becomes colonized [5] and from birth through to 2 years of age the infant gut microbiota is dynamic, unstable and becomes increasingly complex, until it resembles that of an adult. The identity of the first colonizers of the infant gut depends on numerous factors [6], [7] including mode of delivery [8], and pre-term versus full-term gestation [9] and, because of the aforementioned instability of this microbial population, many factors including feeding choice [10], probiotic or prebiotic supplementation [11] and antibiotic exposure [12] can significantly impact on its subsequent development. Indeed, in a previous study, we have demonstrated that the infant microbiota becomes dominated by *Proteobacteria* following ampicillin and gentamicin administration in the first 48 hours of life [13], which may be due to the known high prevalence of antibiotic resistant species within this phylum [14]. Such findings suggest that the infant gut microbiota, though immature and in constant flux, can be a source of resistant bacteria which can become dominant following antibiotic exposure. Given the instability of the infant microbiota in early life, there is considerable opportunity for the infant gut to acquire resistant populations which, if they become established, could have significant effects on shaping the composition of the microbiota later in life [15].

While it is known that antibiotic resistance genes are present within the gut microbiota from early life [4], [16], data with respect to the presence of antibiotic resistance genes in healthy infants with no history of antibiotic treatment is still limited [4]. Furthermore, much of the existing data comes from studies investigating resistance genes in specific commensals, such as strains of *Escherichia coli* or *Lactobacillus* [17], [18], rather than the entire microbiota. Challenges in studying gut microbiota are well documented [6] and include an inability to culture the majority of gut microbes in a laboratory environment [19]. However, metagenomic libraries provide the opportunity to capture metagenomic DNA from complex environments and to functionally screen for phenotypes of interest. The limited existing data available from adult [1], [20] and infant [21], [22] metagenomic banks indicate that human gut microbiota is a source of diverse antibiotic resistance genes. To date, however, there is a significant paucity in infant functional metagenomic studies, particularly relating to infants free from antibiotic treatment. Thus, the aim of this study was to construct a fosmid bank using metagenomic DNA from 6-month-old infants who had never received antibiotic treatment and to screen for the presence of antibiotic resistance genes. By 6 months of age, the infants will have acquired an increasingly complex microbiota and will have had the opportunity to acquire antibiotic resistant strains from numerous sources [5], [23]. Our goal was to provide insight into the infant gut microbiota as a reservoir for resistance genes, when no antibiotic selective pressure exists, using a combination of functional metagenomics and PCR-based approaches. Due to the on-going focus of our research [13], we specifically investigated resistance to aminoglycosides and β-lactam antibiotics. Using both a function-based and function-independent approach, we successfully identified a variety of aminoglycoside and β-lactam resistance genes in the gut microbiota of infants, providing insights into the infant gut resistome.

## Materials and Methods

### Recruitment of volunteers

Infants were recruited as part of the INFANTMET study (http://eldermet.ucc.ie). Parents of infant participants provided written informed consent. Approval for the INFANTMET study was received from the Clinical Research Ethics Committee of the Cork Teaching Hospitals, Cork, Ireland. Infants participating in the study had no antibiotics administered for the first 6 months of life. Fresh faecal samples were collected from 22 6-month-old infants and immediately stored at −80°C until processed.

### DNA extraction

Due to the small volume of each individual sample, it was necessary to pool faecal samples prior to DNA extraction. Faecal samples were homogenized and a 500 mg aliquot from each were pooled to form one sample from which high molecular weight metagenomic DNA was extracted, using a previously described method [24]–[26]. Briefly, faecal samples were homogenized in PBS (Sigma Aldrich, Dublin, Ireland), centrifuged at 1000g×5mins and the supernatant retained. Nycodenz (Axis Shield, UK) density gradient separation was performed to separate out the bacterial cells. Enzymatic digestion of the cells using lysozyme and mutanolysin (Sigma Aldrich) was performed, and subsequently the protein was removed using Proteinase K and ammonium acetate treatment (Sigma Aldrich). DNA was then purified and precipitated using standard chloroform and ethanol precipitation procedures and was eluted in TE buffer.

### Metagenomic bank creation

To allow functional screening of the metagenomic DNA from the infant gut, a fosmid metagenomic bank was created using the EpiCentre CopyControl Fosmid Library Production kit with the pCC1FOS vector, according to the manufacturer's instructions (Cambio, Cambridge, England). Briefly, size selection was performed on the metagenomic DNA using pulse field gel electrophoresis (PFGE) (0.5× TAE; 0.1initial/10 final switch times; 4V; 17 hours; 14°C) and fragments of ∼40 Kb were gel extracted from the low melting point agarose (Promega, Medical Supply Company, Dublin). Fragments were then ligated with the pCC1FOS vector according to the manufacturer's instructions and subsequently packaged into EPI300-T1^R^ *E. coli* plating strain cells. These cells were then plated onto LB agar plates (Difco, Becton, Dickinson & Co, Oxford, England) containing 12.5 µg/ml chloramphenicol, IPTG and Xgal (Sigma Aldrich) and grown overnight aerobically at 37°C. To verify the diversity of the library, random white clones were selected and were digested with *Pst*I and *Nde*I restriction enzymes (New England Biolabs, UK), to determine if different DNA insert sequences were present in our metagenomic bank. The entire library was plated and then picked and stocked in 384-well-format using the QPix2-XT robotic system (Molecular Devices, Berkshire, UK) and was stored at −80°C until screening. A library of ∼220Mb of DNA was created.

### Screening for antibiotic resistant clones

Due to the ongoing focus of our research [13], [27], we chose to concentrate on the resistance of infant gut microbiota to 2 groups of antibiotics, namely the aminoglycosides and β-lactams. For screening of the metagenomic bank, the library was plated onto LB agar with 12.5 µg/ml chloramphenicol and inhibitory concentrations of ampicillin (50 µg/ml) or gentamicin (10 µg/ml) (Sigma Aldrich). These levels of antibiotics were chosen due to their inhibition of host EPI 300-T1^R^ *E. coli* cells. The library was plated in triplicate and plates were incubated aerobically at 37°C for 24–36 hours. Clones found to be resistant over three replicate plates were selected, their resistance phenotype verified by re-streaking onto agar containing the relevant antibiotic and were stocked for further analysis.

### PCR analysis

To investigate which resistance genes were present in the insert DNA that conferred resistance, PCRs were carried out on each of these clones using primers for aminoglycoside and β-lactam resistance genes (Table 1). For the aminoglycosides, we used degenerate primers designed to amplify the acetylation (AAC; *aac* (3)-I, *aac* (3)-II, *aac* (3)-III, *aac* (3)-VI and *aac* (6)), adenylation (ANT; *ant* (2″)-Ia) and phosphorylation (APH; *aph* (2″)-Ic and *aph* (2″)-Id) genes [28], as well as the bifunctional gene *aac* (6′)-*Ie-aph*(2″)-*Ia* [29], [30]. For β-lactam resistance the primer sets for the following genes were used *bla*_TEM_ [31], [32], *bla*_OXA_ [33], *bla*_SHV_ [33], *bla*_ROB_ [32] and *bla*_CTX-M_ [34] (Table 1). Resistant clones were grown overnight in LB broth supplemented with chloramphenicol (12.5 µg/ml) and either ampicillin (50 µg/ml) or gentamicin (10 µg/ml). Fosmids were extracted from the clones using the QIAprep Spin Mini Prep kit (Qiagen, Sussex, UK) and subsequently used as template DNA for PCR analysis. PCRs were performed using previously outlined protocols [28]-[34]. Each reaction contained 25 µl of Biomix Red (MyBio, UK), 1 µl forward primer (10pmol), 1 µl reverse primer (10pmol), fosmid DNA (at the volume required to correspond to 64 ng) from the resistant clone, and PCR grade water (Bioline, Medical Supply Company, Dublin, Ireland) to a final reaction volume of 50 µl. All reactions were performed in duplicate. PCR products were visualized using gel electrophoresis (1.5% agarose, 1× TAE, 100V). Negative controls, in which the DNA template was substituted with PCR-grade water, failed to generate an amplicon, ruling out the possibility of contamination. Successful duplicate PCRs were pooled and cleaned using AMPure (Beckman Coulter UK) magnetic bead-based purification procedures.

**Table 1:** Primers used in this study.

Target gene family	Primer name	Sequence 5′-3′	Annealing temperature °C	Ref.
**β-lactamase genes**				
***bla*** **_TEM_**	RH605	TTTCGTGTCGCCCTTATTCC	60	Bailey *et al.* (2011)
	RH606	CCGGCTCCAGATTTATCAGC		
	Bla_TEMF	TGGGTGCACGAGTGGGTTAC	57	Tenover *et al.* (1994)
	Bla_TEMR	TTATCCGCCTCCATCCAGTC		
***bla*** **_ROB_**	Bla_ROBF	ATCAGCCACACAAGCCACCT	62	Tenover *et al.* (1994)
	Bla_ROBR	GTTTGCGATTTGGTATGCGA		
***bla*** **_SHV_**	Bla_SHVF	CACTCAAGGATGTATTGTG	58	Briñas *et al.* (2002)
	Bla_SHVR	TTAGCGTTGCCAGTGCTCG		
***bla*** **_OXA_**	Bla_OXAF	TTCAAGCCAAAGGCACGATAG	64	Briñas *et al.* (2002)
	Bla_OXAR	TCCGAGTTGACTGCCGGGTTG		
***bla*** **_CTX-M_**	Bla_CTX-MF	CGTTGTAAAACGACGGCCAGTGAATGTGCAGYACCAGTAARGTKATGGC	55	Monstein *et al.* (2009)
	Bla_CTX-MR	TGGGTRAARTARGTSACCAGAAYCAGCGG	60	
**AG resistance genes**				
***aac*** ** (3)-I**	Faac3-1	TTCATCGCGCTTGCTGCYTTYGA	58	Heuer *et al.* (2002)
	Raac3-1	GCCACTGCGGGATCGTCRCCRTA		
***aac*** ** (3)-II/VI**	Faac3-2	GCGCACCCCGATGCMTCSATGG	58	
	Raac3-2	GGCAACGGCCTCGGCGTARTGSA		
	Facc3-6	GCCCATCCCGACGCATCSATGG		
	Raac3-6	CGCCACCGCTTCGGCATARTGSA		
***aac*** ** (6**′**)-II/Ib**	Faac6	CACAGTCGTACGTTGCKCTBGG	58	
	Raac6	CCTGCCTTCTCGTAGCAKCGDAT		
***ant*** ** (2**″**)-Ia**	Fant	TGGGCGATCGATGCACGGCTRG	58	
	Rant	AAAGCGGCACGCAAGACCTCMAC		
***aph*** **(2**″**)-I**	Faphc	CCCAAGAGTCAACAAGGTGCAGA	55	
	Faphd	GGCAATGACTGTATTGCATATGA	55	
	Raph	GAATCTCCAAAATCRATWATKCC		
***aac*** **(6**′**)-** ***Ie*** **-** ***aph*** **(2**″**)-** ***Ia***	aac-aphF	GAGCAATAAGGGCATACCAAAAATC	47	De Fatíma Silva Lopes *et al.* (2003)
	aac-aphR	CCGTGCATTTGTCTTAAAAAACTGG		
	aac6-aph2F	CCAAGAGCAATAAGGGCATACC	55	Schmitz *et al*. (1999)
	aac6-aph2R	CACACTATCATAACCATCACCG		

AG: aminoglycoside.

To determine if additional resistance genes were present in the infant gut microbiome that had not been captured in the metagenomic bank, additional PCRs for detecting resistance genes were performed on the metagenomic DNA which was used for construction of the fosmid bank. PCR analysis was performed using the primers and protocols outlined above. Following PCR amplification, amplicons were cloned using the TOPO TA cloning kit (Invitrogen, Dublin, Ireland) according to the manufacturer's instructions. TOPO cloning reactions were then transformed into *E. coli* TOP 10 cells and plated on LB agar containing antibiotics for the selection of the cloning vector (either kanamycin 50 µg/ml or ampicillin 50 µg/ml). Plasmids were then extracted from overnight cultures of the TOPO sub-clones using the QIAprep Spin Mini Prep kit (Qiagen, Sussex, UK) to facilitate subsequent DNA sequencing.

### Sequencing and analysis

A subset of PCR products from the resistant fosmid clones were sent for Sanger sequencing to determine their closest homologue (Source Biosciences, Dublin, Ireland). The subset included representatives from each of the genes investigated and from clones containing multiple resistance genes. Plasmid DNA from a subset of the TOPO sub-clones of the metagenomic DNA PCR products were also sent for Sanger sequencing. Sequencing reads were BLASTed against the NCBI non-redundant database using BLASTx (http://blast.ncbi.nlm.nih.gov/). In the event where multiple hits occurred, the BLAST hit which displayed greatest homology (based on E value) is reported.

### Minimum inhibitory concentration assays

Minimum inhibitory concentration (MIC) tests were conducted on gentamicin (Gent^R^) and ampicillin resistant (Amp^R^) fosmid clones to determine the level of resistance conferred by the insert DNA. In order to determine the relative increase in resistance, MICs were compared to the control strain (EPI300-T1^R^ *E. coli* + empty pCC1FOS fosmid). MICs were performed according to the British Society for Antimicrobial Chemotherapy guidelines [35]. An antibiotic range of 256–0.25 mg/L was used. Positive (broth + inoculum only) and negative controls (broth + antibiotic only) were included in each assay. Plates were incubated aerobically at 37°C for 24 hours. The MIC was determined as the lowest concentration of antibiotic at which no visible growth occurred.

## Results

### Identification of gentamicin and ampicillin resistant clones within the infant gut microbiome using a functional metagenomic bank

**Figure 1:**
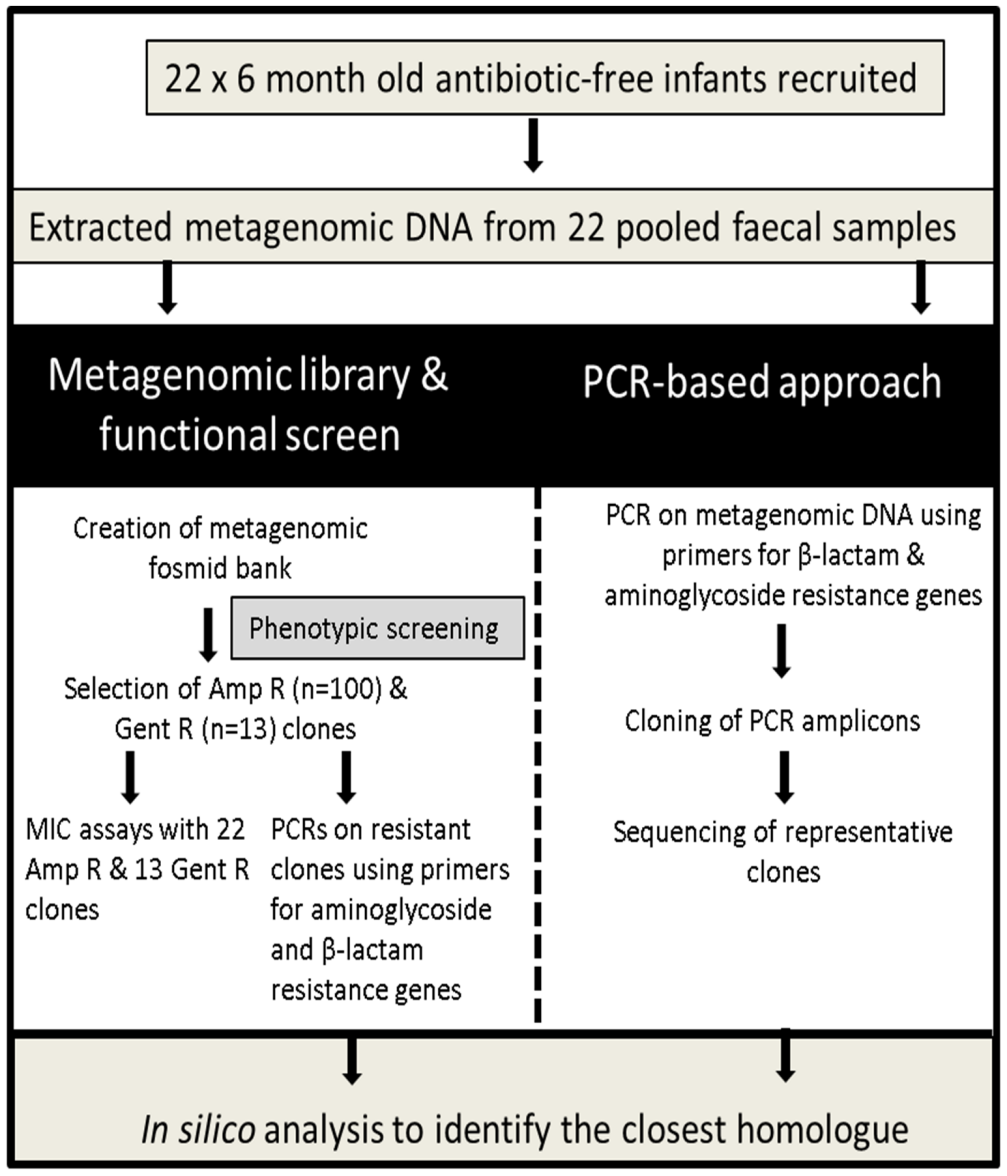
Schematic of the approach applied in this study.

Ten white clones were picked at random from LB agar plates containing 12.5 µg/ml chloramphenicol, IPTG and X-gal and underwent restriction digestion using *Pst*I and *Nde*I restriction enzymes. These randomly selected clones had unique restriction profiles (data not shown), establishing that the metagenomic library contained different insert DNA, demonstrating a diverse metagenomic fosmid bank had been successfully created. Our library contained ∼220Mb of metagenomic DNA, which contained 0.45 ampicillin resistant hits/Mb and 0.059 gentamicin resistant hits/Mb. To determine which genes were responsible for the resistance in the isolated clones, PCR analysis was conducted on each of the resistant clones using primers for β-lactam and aminoglycoside resistance genes (sequencing files available at http://dx.doi.org/10.6070/H46971H6). Furthermore, to determine if additional genes could be detected in the uncloned metagenomic DNA, PCR analysis was completed on this DNA with the same degenerate primers as used on the resistant clones (Figure 1).

### PCR and Sanger sequencing of β-lactam resistant clones

Analysis of the one hundred Amp^R^ clones using *bla*_TEM_ primers, revealed that they all contained *bla*_TEM_ genes (Table 2). Based on sequencing analysis, these genes shared closest homology with *bla*_TEM_ genes from uncultured soil bacteria (clones 2, 3, 4, 5, 6, 10, 16, 17, 123, 128), *Shigella* sp. (clone 4), *Serratia marcescens* (clone 126) and *Citrobacter freudii* (clones 17 and 18) (Table 2). Sequencing of amplicons generated using the same *bla*_TEM_ primers and uncloned metagenomic DNA, revealed a number of different *bla*_TEM_ genes that shared closest homology with genes from *Klebsiella pneumoniae*, *E. coli*, *Salmonella enterica* and *Staphylococcus aureus* (Table 3).

**Table 2:** Identity of ampicillin and gentamicin resistant genes amplified from the infant gut metagenomic bank.

Accession number	Primer name	Clones in which genes detected	Closest homologue*	E value	% ID
**ROB**	Bla_ROBF				
	Bla_ROBR				
WP_016930608.1		15,50,51,61,62,63	Hypothetical protein *S. haemolyticus*	1e^−20^	53
**TEM**	RH605				
	RH606				
	Bla_TEMF				
	Bla_TEMR				
AGH19654.1		2, 5,6	β-lactamase partial uncultured soil bacterium	1e^−134^	100
AGH19657.1		3,16,17	β-lactamase partial uncultured soil bacterium	4e^−139^	99
AAP93842.1		17,18	β-lactamase *C. freudii*	5e^−138^	99
AEN75339.1		4	β-lactamase TEM *Shigella* sp.	4e^−141^	99
AGH19655.1		2,123	β-lactamase uncultured soil bacterium	2e^−97^	100
AGH19650.1		3,4,10,128	β-lactamase uncultured soil bacterium	7e^−97^	100
AAP93841.1		126	β-lactamase *S. marcescens*	2e^−97^	98
**CTX-M**	Bla_CTX-MF				
	Bla_CTX-MR				
AEZ49563.1		2,3	β-lactamase CTX-M-1 *E. coli*	2e^−116^	99
BAD16611.1		4,136	β-lactamase CTX-M-36 *E. coli*	1.10e^−127^	99
AAB22638.1		140,145	β-lactamase penicillin amido β-lactamase hydrolase *E. coli*	2e^−128^	100
**OXA**	Bla_OXAF				
	Bla_OXAR				
AGN75112.1		140	TEM-190 β-lactamase *E. coli*	2e^−04^	38
ADZ11076.1		132	β-lactamase TEM *E. coli*	2e^−40^	71
***ant*** ** (2″)-Ia**	Fant				
	Rant				
YP_005176240.1		3,7,9,10,11,12,33,34,36	AG 2 O′ adenyltransferase *P. multocida*	1e^−79^	96
WP_000292466.1		8	AG adenyltransferase *E. coli*	5e^−75^	96
***aph*** ** (2″)-Id**	Faphd				
	Raph				
WP_0214010241.1		5, 6, 8,11,12,13, 34,	aph *C. difficile*	4e^−95^	100
***aac*** **(6)**	Faac6				
	Raac6				
AAA25680.1		34,36	AG 6' N acetyltransferase *P. fluorescens*	5e^−33^	97
***aac*** ** (6′)-** ***Ie*** **-** ***aph*** ** (2″)-** ***Ia***	aac-aphF				
	aac-aphR				
	aac6-aph2F				
	aac6-aph2R				
WP_010714603.1		3,4,5,6,11	bifunctional *aac* (6′)-*Ie*-*aph* (2″) *E. faecalis*	1e^−96^	100
AFR11868.1		4,8,13	bifunctional 6' AG N acetyltransferase/2'' AG phosphotransferase *S. epidermidis*	8e^−32^	98
AFM29914.1		5	Gentamicin resistance protein *Enterococcus* sp.	3e^−30^	96
WP_010782592.1		10	Bifunctional AAC/APH *E. faecium*	1e^−31^	98

* Result presented is the closest homologue to our sequence (based on lowest E value).

**Table 3:** Sequencing results from TOPO cloning of ampicillin and gentamicin resistant genes from metagenomic DNA.

Accession #	Closest homologue*	E value	% ID
**TEM**			
AAL03985.1	ESBL TEM-71* K. pneumoniae*	7e^−154^	99
WP_004207849.1	β-lactamase TEM partial *K. pneumoniae*	3e^−153^	97
WP_017431996.1	β-lactamase partial *S. aureus*	1e^−112^	84
WP_019405145.1	β-lactamase partial *K. pneumoniae*	6e^−154^	100
AEN02824.1	β-lactamase TEM-1 *K. pneumoniae*	4e^−111^	99
ADE18896.1	TEM-1 β-lactamase *S. enterica*	7e^−112^	97
ABG46354.1	ESBL *E. coli*	1e^−139^	100
AEN02826.1	β-lactamase TEM-1 *K. pneumoniae*	1e^−108^	99
**CTX-M**			
AAB22638.1	β-lactamase penicillin amido β-lactam hydrolase *E. coli*	1e^−139^	100
AEZ49551.1	β-lactamase CTX-M-1 *K. pneumoniae*	5e^−129^	99
AEZ49563.1	β-lactamase CTX-M-1 *E. coli*	8e^−140^	97
ABG46356.1	ESBL *K. pneumoniae*	1e^−138^	99
***ant*** ** (2″)-Ia**			
YP_005176240.1	AG 2 O adenyltransferase *Pasteurella multocida*	1e^−95^	99
WP_000946493.1	2 AG nucleotidyltransferase *A. baumannii*	3e^−97^	99
***aph*** ** (2″)-Id**			
AAW59417.1	*E. faecium* aph 2 Id	5e^−98^	90
3Sg8_9	Crystal structure of AG 2'' phosphotransferase Tobramycin resistance gene	5e^−109^	100
WP_021401024.1	aph (2″)-Id *C. difficile*	6e^−108^	98
***aac*** ** (6′)-** ***Ie*** **-** ***aph*** ** (2″)-** ***Ia***			
WP_ 001028140.1	GNAT family acetyltransferase *S. aureus*	2e^−109^	100
AAX82584.1	Bifunctional AG modifying enzyme *E. faecalis*	2e^−106^	99
WP_002417297.1	Phosphotransferase enzyme family protein *E. faecalis*	4e^−113^	99

* Result presented is the closest homologue to our sequence based on lowest E value.

ESBL: extended spectrum β-lactamase.

AG: aminoglycoside.

PCRs revealed that half of the Amp^R^ clones contained *bla*_CTX-M_ genes (including CTX-M-1 and CTX-M-36 gene homologues), which shared homology with 1 of 3 different *bla*_CTX-M_ genes present in *E. coli*. Sequencing of *bla*_CTX-M_ PCR products generated directly from metagenomic DNA identified additional sources of these genes, detecting genes that shared homology with *bla*_CTX-M_ genes present in *E. coli* (including one source that was not detected in the analysis of the Amp^R^ clones) and *K. pneumoniae* (Table 3).

Just 2 of the 100 Amp^R^ clones gave PCR products using the *bla*_OXA_ primers, which surprisingly resembled 2 different *bla*_TEM_ genes, rather than *bla*_OXA_ genes, from a variety of *E. coli*. No *bla*_OXA_ genes were amplified when PCR was directly applied to the metagenomic DNA from the infant faecal samples. Six of the 100 Amp^R^ clones (clones 15, 50, 51, 61, 62 and 63) contained *bla*_ROB_ genes and all shared closest homology (53% identity, 40% query cover) with a hypothetical protein from *Staphylococcus haemolyticus*. Direct PCR amplification using template metagenomic DNA failed to reveal any *bla*_ROB_ genes. Finally, *bla*_SHV_ primers did not generate amplicons from either the Amp^R^ clones or uncloned metagenomic DNA.

### PCR and Sanger sequencing of aminoglycoside resistant clones

Degenerate PCR primers were employed to determine whether the fosmids conferring gentamicin resistance contained homologues of known aminoglycoside resistance genes. Acetylation, adenylation and phosphorylation genes were detected in the Gent^R^ clones (Table 2). With respect to aminoglycoside acetylation-associated genes, *aac* (3)-I, *aac* (3)-II, *aac* (3)-III and *aac* (3)-VI were not detected in either the 13 Gent^R^ clones or in the uncloned metagenomic DNA. However, all 13 Gent^R^ clones contained an *aac* (6) gene that most closely resembled a *Pseudomonas fluorescens* gene. Interestingly, the *aac* (6) primers did not generate amplicons when used directly with metagenomic DNA (Table 3). Aminoglycoside adenylation, *ant* (2″)-Ia, genes were detected in 9 of the 13 Gent^R^ clones (Table 2). Eight of these 9 Gent^R^ clones shared closest homology with aminoglycoside 2′ O adenyltransferase genes found in *Pasteurella multocida*, while that from the remaining clone resembled an *E. coli* associated gene. Similar sources of these genes were also detected when PCR was performed with the *ant* (2″)-Ia primers and uncloned metagenomic template DNA (Table 3). Following cloning and sequencing of these amplicons, it was apparent that 4 of the 5 clones contained *ant* (2″)-Ia genes sharing closest homology (99% identity, 97% query cover) with genes present in *P. multocida* (identical to the source of these genes detected in the Gent^R^ clones). The fifth clone that was sequenced contained insert DNA sharing closest homology with a nucleotidyltransferase from *A. baumannii* (99% identity, 97% query cover).

When primers were applied to investigate the presence of aminoglycoside phosphorylation genes using *aph* (2″)-Id primers, 10 of the 13 Gent^R^ clones contained these genes (Table 2). A number of these Gent^R^ clones (5, 6, 8, 11, 12, 13 and 34) shared closest homology with phosphorylation genes from *Clostridium difficile* (100% identity and 94% query cover). When these *aph* (2″)-Id primers were used directly with metagenomic DNA, genes sharing homology with *C. difficile* (identical to those detected in the Gent^R^ clones), *Enterococcus faecium* and an unknown source of *aph* (2″)-Id genes were detected (Table 3).

Finally, using 2 different primer sets, it was found that all Gent^R^ clones contained the bifunctional gene *aac*(6′)-*le*-*aph*(2″)-*Ia*. These genes shared homology with bifunctional *aac*(6′)-*le*-*aph*(2″)-*Ia* genes from *Enterococcus faecalis* (clones 3, 4, 5, 6 and 11), *Streptococcus epidermidis* (clones 4, 8 and 13), *E. faecium* (clone 10) and *Enterococcus* sp. (clone 5) (Table 2). Sequencing of the amplicons generated with uncloned metagenomic DNA, identified genes sharing closest homology with bifunctional genes from *E. faecalis* (which were distinct from those detected in the Gent^R^ clones) and from *S. aureus* (WP_001028140.1), a source which was not detected using PCR analysis on the Gent^R^ clones (Table 3).

### MIC analysis of ampicillin and gentamicin resistant clones

To determine the level of resistance conferred by the fosmid-cloned metagenomic DNA to ampicillin or gentamicin, MIC assays were conducted. A representative subset of 22 of the 100 Amp^R^ clones (including those containing genes only detected in a limited number of clones e.g. *bla*_OXA_ and clones containing multiple β-lactam resistance genes) and all 13 of the Gent^R^ clones were studied. MICs were compared to the host EPI 300-T1^R^ *E. coli* cells containing the empty pCC1FOS fosmid.

The Amp^R^ clones exhibited a 2- (clone 6) to 512- (clone 2) fold increase in resistance to ampicillin compared to the empty fosmid control (Table 4). The large variation in MICs between the Amp^R^ clones highlighted the fact that different, but related, genes conferred different levels of resistance to the host *E. coli* cells, most likely due to differences in expression/translation in the surrogate *E. coli* host. Clones containing multiple β-lactam resistance genes (clones 2, 3, 4, 62, 128, 136 and 140), including *bla*_TEM_, *bla*_CTX-M_ and *bla*_OXA_ genes, were found to confer the highest levels of resistance to β-lactams. In contrast, when MICs were conducted on the 13 Gent^R^ clones, all clones showed similar levels of resistance to gentamicin with MICs of 4-8 fold higher than that of the control (Table 5).

**Table 4:** MICs of ampicillin resistant clones.

Amp^R^ clone	MIC fold difference compared to the control
2	512
3	128
4	256
5	4
6	2
10	16
15	4
16	32
17	64
18	32
50	4
51	16
61	64
62	256
63	4
123	64
126	16
128	128
132	16
136	256
140	128
145	8

**Table 5:** MICs of gentamicin resistant clones.

Gent^R^ clones	MIC fold difference compared to the control
3	8
4	8
5	8
6	8
7	8
8	8
9	8
10	8
11	8
12	8
13	4
33	4
34	8

## Discussion

The infant gut microbiota is in constant flux during the first two years of life [23]. During this time there is considerable opportunity for the infant to acquire antibiotic resistant populations. Given the opportunity, resistant populations could become dominant in the gut following antibiotic therapy or may contribute to antibiotic resistance gene dissemination. In this study we set out to use a metagenomic fosmid bank to functionally screen the gut microbiota of 22 healthy infants who had not been treated with antibiotics, for antibiotic resistance genes. Additionally, we wanted to perform PCR analysis directly on uncloned metagenomic DNA to determine if additional genes would be detected, that had not been captured in the functional bank. Using such an approach, we have demonstrated that the infant gut is a source of a variety of genes encoding resistance to aminoglycosides and β-lactams. One hundred Amp^R^ clones were detected in the gut microbiota of infants through screening of the metagenomic fosmid bank. Applying a PCR-based approach to determine the resistance genes present in the resistant clones, we detected *bla*_TEM_, *bla*_OXA_, *bla*_ROB_ and *bla*_CTX-M_ genes. Interestingly, the *bla*_TEM_ genes detected exhibited greatest homology with genes from uncultured bacteria. Such results highlighted the ability of our strategy to reveal genes from more cryptic sources, including β-lactam resistance genes from sources not accessible through classical culture-based approaches. It was also notable that we detected such a prevalence of ampicillin resistance genes, and that they appear to have originated from a diverse range of species. Nonetheless, the majority of the β-lactam resistance genes identified shared homology with β-lactam resistance genes present in *E. coli*, *Shigella* and *Serratia*, which are common sources of such genes [36]–[38]. When PCRs were performed using *bla*_OXA_ primers, just 2 of the 100 Amp^R^ clones were found to contain such genes. However, sequencing of these amplicons revealed that they shared greatest homology with *bla*_TEM_ genes (Table 2). Therefore, it appears that in this instance, the infant gut microbiota did not contain *bla*_OXA_ genes and in their absence, the *bla*_OXA_ primers enabled the detection of *bla*_TEM_ homologues. This is not entirely unexpected, given previous observations of 3 sites of homology between the amino acid sequence of the OXA 2 gene and TEM β-lactamases [39].

This metagenomic study has successfully identified acetylation, adenylation and phosphorylation genes conferring aminoglycoside resistance in the infant gut microbiota. The fact that these genes resemble genes from bacteria such as *E. coli*, *E. faecalis*, *S. epidermidis* and *C. difficile* is not surprising given that a number of the observed resistance genes are resident in such species [30], [40], [41]. A recent study which employed a metagenomic approach to investigate antibiotic resistance in infants, also detected a high prevalence of aminoglycoside resistance genes [22]. In that instance, they too identified aminoglycoside acetylation, adenylation and phosphorylation genes in the infant gut which shared homology with *C. difficile* and *E. faecium*. As these bacteria may be among the first colonizers of the infant gut, these findings support previous research which suggests antibiotic resistance and the antibiotic resistome is established in early life, perhaps even from birth, irrespective of antibiotic exposure, and is closely associated with microbes from the maternal and environmental sources it is exposed to during and immediately after birth [4]. This is consistent with the apparently ubiquitous nature of antibiotic resistance genes in the environment, with studies having demonstrated that antibiotic resistant isolates can be detected in individuals from remote areas of the world who lack, or who have minimal, antibiotic exposure [42]–[44].

In addition to screening the fosmid metagenomic bank, a PCR-based analysis of uncloned metagenomic DNA was also carried out. Previous studies demonstrated the success of such an approach with respect to detecting genes that were present at a low level and had not been captured in the metagenomic fosmid bank [21]. Additionally, this approach also addresses situations whereby certain genes may not be expressed or their products may not be functional in *E. coli*, e.g. as previously shown in the case of *strA* and *strB* [45]. However, previous studies have demonstrated that genes from at least 4 major phyla from the gut microbiome can be functional in *E. coli* hosts [46]. It is also possible to create metagenomic banks using Gram-positive hosts such as *Streptomyces* to identify genes which may not be expressed in Gram-negative hosts [47]. In our study, as our library was only made in a Gram-negative host, we conducted PCR analysis directly on uncloned metagenomic DNA, with the aim of identifying additional resistance genes to those captured in our functional screen. Thus, in this instance, we have demonstrated the usefulness of supplementing functional metagenomics with a PCR-based approach directly on metagenomic DNA to enable detection of antibiotic resistance genes apparently originating from different species than those that were identified during the course of the functional screen. In the majority of cases the putative sources of genes detected by PCR with metagenomic library isolates and with uncloned metagenomic template DNA were similar, though in some circumstances additional insights were provided using this combined approach. For example, some *bla*_TEM_ genes identified from the functional screen shared closest homology with genes from uncultured soil bacterium, whereas direct PCR-screening highlighted homologues of genes found in *K. pneumoniae*, *S. aureus*, *S. enterica* and *E. coli*. Furthermore, in certain instances resistance genes were captured by the metagnomic bank (*aac* 6) that were not detected when PCR analysis was completed directly on metagnomic DNA. This demonstrates that combining different experimental approaches allows for the most comprehensive insight into the gut microbiota as a source of antibiotic resistance genes. It should also be noted that, if screened for, we expect that our metagenomic library would also uncover resistance genes for other major families of antibiotics. Multi-drug resistant Gram-positive bacteria represent a serious threat to human health [48]. Several recent functional metagenomic studies screened the gut microbiota of children for resistance against antibiotics which specifically target Gram-positive bacteria (including fosfomycin and tigecycline) [20], [22]. In the study of Moore *et al*., clones resistant to antibiotics which target Gram-positive bacteria were detected [22], demonstrating that the gut microbiota of children and teenagers is a reservoir for resistant Gram-positive bacteria. Thus, while our focus was resistance to aminoglycoside and β-lactam antibiotics, future screening of this metagenomic bank for resistance to other antibiotics would likely yield positive clones.

This study is one of a limited number that has demonstrated the existence of antibiotic resistant genes in the healthy infant gut microbiota using a metagenomic library approach. Although used to investigate antibiotic resistance in environments as diverse as gull faeces [49] or soil from apple orchards [45], to date the use of metagenomic banks to screen the infant gut microbiota for resistance genes has been limited [21], [22]. In particular, our study adds considerably to current data on the infant gut resistome by supporting and supplementing existing results [22], which have demonstrated that the infant gut is a reservoir for antibiotic resistance. By studying 6-month-old infants with no previous antibiotic exposure, we were able to establish a comprehensive insight into the resistome of infants devoid of antibiotic selective pressure. Our results supplement those of Moore *et al*. who investigated antibiotic resistance in children and teenagers using functional metagnomics [22]; however, in that instance they examined antibiotic resistance across a wide age range (1 month to 19 years of age), while our study provides a comprehensive insight into the infant resistome specifically at 6 months of age, demonstrating the early development of this resistome even without treatment with antibiotics. Additionally, we have demonstrated the benefits of supplementing functional screening with PCR and MIC assays, which enables the complexity of the infant resistome to be fully appreciated.

The approach applied in our study has both strengths and limitations. The strengths lie in the combination of approaches employed (functional metagenomics, PCR and MICs) to provide insights into the infant resistome. Whilst functional metagenomics enabled the detection of genes from apparently more novel sources (including uncultured soil bacteria), PCR analysis using uncloned metagenomic DNA as a template, detected a number of gene(s) from additional sources not captured by our functional analysis. Moreover, MIC analysis highlighted the wide range of increased resistance to aminoglycosides and β-lactams conferred by each of the clone inserts. The limitations are that even using a combined metagenomic approach, we were unable to detect novel aminoglycoside or β-lactam resistance genes. It would be useful, therefore, to construct an additional infant metagenomic library using a Gram-positive host, as well as in *E. coli*, to investigate whether different resistance genes could be captured and expressed. Furthermore, it would be interesting to determine expression levels of antibiotic resistance genes harboured by the infant gut microbiota. We hope to apply such approaches in future related studies.

The findings from our study are important for several reasons. Firstly, as the infant gut is populated through complex bacterial acquisitions from maternal, dietary and environmental sources, there is the potential to control the populations colonizing infants, i.e. to maximize colonization with those bacteria that may be beneficial and pose less risk of contributing to the antibiotic resistome. Additionally, in cases where antibiotic administration is required, the route of antibiotic administration should be considered carefully when different options are available. Indeed, research using a murine model has demonstrated that antibiotic administration through intravenous injection resulted in significantly less, or the delayed development of, antibiotic resistant genes in gut microbiota populations compared to oral administration [50]. Although outside the scope of the present study, it would also seem that further investigations with respect to links between maternal antibiotic exposure and subsequent antibiotic resistant populations in the infant would be beneficial. In one previous instance, tetracycline resistance (tet^R^) in the gut microbiota of one mother-infant pair was studied [21]. More specifically, resistance genes from the gut microbiota of a vaginally delivered, exclusively breastfed male infant, one month after birth, were compared with those within the gut microbiota of his mother. Among the findings was an observation of some identical resistance genes in both populations, suggesting that the transfer of antibiotic resistant populations from the mother to the infant may occur, potentially through maternal microbiota transmission during delivery, physical contact or through breastfeeding [21]. Others have also demonstrated shared antibiotic resistance gene pools between mother and infant faecal samples [4], which may be associated with resistance genes detected in breast milk. Thus, it is likely that infant gut microbiota acquired during delivery and breastfeeding can contribute to the development of the infant resistome.

In our study, the gut microbiomes of 6 month-old infants, who had not previously been treated with antibiotics, harbored diverse antibiotic resistance genes. Metagenomic fosmid bank creation allowed functional screening for resistance genes in the gut microbiota of infants and revealed genes from diverse sources including those previously linked with uncultured bacteria. Combining this sophisticated approach with PCR techniques, provided a particularly unique insight into the presence of resistance genes in the infant gut microbiota. The results not only act as a cautionary insight into the prevalence of antibiotic resistance in infants, even in cases where no antibiotic selective pressure occurred, but also demonstrate the power of combining several metagenomic approaches to provide important insights into the infant gut resistome.
